# Active Biohybrid Nanocomposite Films Made from Chitosan, ZnO Nanoparticles, and Stearic Acid: Optimization Study to Develop Antibacterial Films for Food Packaging Application

**DOI:** 10.3390/ma16030926

**Published:** 2023-01-18

**Authors:** Nugraha Edhi Suyatma, Sanjaya Gunawan, Rani Yunia Putri, Ahmed Tara, Fazilay Abbès, Dwi Yuni Hastati, Boussad Abbès

**Affiliations:** 1 Department of Food Science and Technology, Faculty of Agricultural Engineering and Technology, IPB University, Bogor 16880, Indonesia; 2 MATIM, UFR Sciences Exactes et Naturelles, Université de Reims Champagne-Ardenne, Campus Moulin de la Housse, 51100 Reims, France; 3 Food Quality Assurance, College of Vocational Studies, IPB University, Bogor 16128, Indonesia

**Keywords:** biohybrid nanocomposite films, antibacterial films, ZnO nanoparticles, full factorial optimization, food packaging, chitosan film

## Abstract

Chitosan is a biopolymer with great potential as food packaging due to its ability to create a film without additives and its better mechanical and antibacterial qualities compared to other biopolymers. However, chitosan film still has limitations due to its high moisture sensitivity and limited flexibility. Incorporating ZnO nanoparticles (ZnO-NPs) and stearic acid (SA) into chitosan films was expected to improve tensile strength, water vapor barrier, and antibacterial capabilities. This study aims to find the optimal formula for biohybrid nanocomposite films composed of chitosan, ZnO-NPs, and SA. The full factorial design approach—4 × 2 with 3 replicates, i.e., two independent variables, namely %ZnO-NPs at 4 levels (0%, 0.5%, 1%, and 3%, *w*/*w*) and %SA at 2 levels (0% and 5%, *w*/*w*)—was utilized to optimize chitosan-based biohybrid nanocomposite films, with the primary interests being antibacterial activities, water vapor barrier, and tensile strength. The incorporation of ZnO-NPs into chitosan films could increase antibacterial activity, while SA decreased it. The addition of SA had a good effect only in decreasing water vapor transmission rate (WVTR) values but a detrimental effect on other film properties mentioned above. The incorporation of ZnO-NPs enhanced all functional packaging properties of interest. The suggested solution of the optimization study has been validated. As a result, the formula with the inclusion of 1% ZnO-NPs without SA is optimal for the fabrication of active antibacterial films with excellent multifunctional packaging capabilities.

## 1. Introduction

Food contact materials are materials and articles that are intended for direct contact with food, such as containers and packaging. These can be created from plastic, rubber, paper, and metal [1]. Plastic is the most used material for food packaging since it is flexible, easy to mold, heat sealable, lightweight, tough to break, and inexpensive to make. Thus, over 50% of packaged food in Europe is packed in plastics [2], and the packaging sector has become Europe’s largest end-use market for plastic [3]. However, many plastics are single-use, which, when combined with low recycling or reuse rates, contributes significantly to environmental pollution [4]. Plastic packaging’s environmental consequences have inspired research in eco-friendly packaging materials, such as polysaccharides, proteins, and lipids.

Thanks to their ability to form films, nontoxicity, low cost, strong mechanical and gas barrier properties, accessibility, and biodegradability [5,6], polysaccharides are the most often-utilized material. Due to its chemical-free film production and antibacterial properties, chitosan has been the subject of many research studies on different polysaccharides seeking to create active antimicrobial food packaging. Chitosan has been used in numerous foods as a natural preservative, but its poor mechanical resistance, water resistance, and barrier properties have limited its application [7]. To avoid these drawbacks, it is conceivable to create biohybrid nanocomposite films by reinforcing chitosan with nanosized fillers [8].

Biohybrid nanocomposite is defined as a bio-based polymer loaded with inorganic nanoparticles, such as metal oxides, clays, and silica [9]. The incorporation of inorganic nanoparticles into bio-based polymers as food contact materials intends to enhance the mechanical and barrier properties of the polymers while also offering an active function. ZnO-NPs are the most promising inorganic nanoparticles due to their amazing physical and chemical characteristics. Recently, Aji et al. [10] conducted a meta-analysis on the evaluation of numerous factors of ZnO-NPs on bionanocomposite film properties. According to the current meta-analysis, using ZnO-NPs as nanofillers in bionanocomposite films significantly enhanced tensile strength, elongation at break, and water vapor permeability (WVP). In addition, this study demonstrated that the optimal film characteristics for increasing the shelf-life of food products could be achieved by deploying ZnO-NPs with proper specifications.

ZnO-NPs are nontoxic inorganic metal oxides widely applied in the food industry as zinc supplements and antibacterial agents, particularly in edible coatings, to prevent food spoilage by fungi and bacteria. The incorporation of ZnO-NPs could increase the antibacterial activity of the packaging system for extending the shelf-life of foods packaged with these materials. The United States Food and Drug Administration (FDA) has categorized ZnO as generally recognized as a safe substance (GRAS) [11,12] since it is nontoxic to human cells. Siddiqi et al. [13] evidenced that ZnO-NPs with concentrations exceeding 100 g/mL were harmful, but ZnO-NPs with concentrations below 100 g/mL were safe. In addition, Jayasuriya et al. [14] discovered that composite films containing 1% ZnO-NP by weight (particle size about 30 nm) revealed low cytotoxicity of cells, but ZnO-NP concentrations larger than 5% exhibited evident toxicity. Several studies have shown that biohybrid chitosan-ZnO-NP nanocomposite films are superior for retaining fruit color, minimizing water loss, enhancing antibacterial activity, and extending the freshness period. Li et al. [8] and Rahman et al. [15] showed that chitosan – ZnO-NPs composite films could extend the shelf life of cherry tomatoes and raw beef, respectively.

Stearic acid (SA) is a saturated fatty acid known for its benefit on water vapor barrier properties. It has been commonly incorporated into biopolymer-based films, such as starch [16,17,18], hydroxypropyl methylcellulose [19], and gelatin [20], to increase the water vapor barrier. Casillas-Vargas et al. [21] found that SA has antibacterial activity against gram-positive and gram-negative microorganisms. However, the antibacterial activity of SA in biocomposite films has not been investigated yet. Thus, the combination of ZnO-NPs and SA can enhance several functional packaging properties, such as the water vapor barrier, mechanical properties (higher tensile strength and elasticity simultaneously), and antibacterial properties of chitosan-based biohybrid nanocomposite films.

This work aimed to find the optimal formulation for producing chitosan, ZnO-NP, and SA biohybrid nanocomposite films. The best combination of mechanical, water vapor transmission rate and antibacterial properties against gram-negative and gram-positive bacteria was determined using a full factorial design (FFD) during the optimization phase. Differential scanning calorimetry (DSC), Fourier-transform infrared (FTIR) spectroscopy, scanning electron microscopy (SEM), and other physical characterization techniques were also used to investigate representative samples of biohybrid nanocomposite films in this study. This research could provide the optimal formulation and guidance for preparing biohybrid nanocomposite films, which could be used as active antibacterial films or coatings for food packaging applications.

## 2. Materials and Methods

### 2.1. Materials

The materials used for producing nanocomposite films/coatings were chitosan powder with a 90.2% degree of deacetylation (measured via colloid titration) from Biotech Surindo (Cirebon, Indonesia), Glycerol 99% Sigma Aldrich, Darmstadt, Germany), ZnO-NPs with an average particle size of 20 nm and 95% purity from Wako Pure Chemicals (Osaka, Japan), and lactic acid 90% (Sigma Aldrich, Darmstadt, Germany). Nutrient agar and nutrient broth (Merck KGaA, Darmstadt, Germany) were employed for the antimicrobial activity test; test cultures taken from the collection of SEAFAST CENTER IPB University were *Bacillus cereus* (ATCC 11778), *Escherichia coli* (ATCC 25922), and *Staphylococcus aureus* (ATCC 25923).

### 2.2. Nanocomposite Film/Coating Solution Preparation

**Figure 1:**
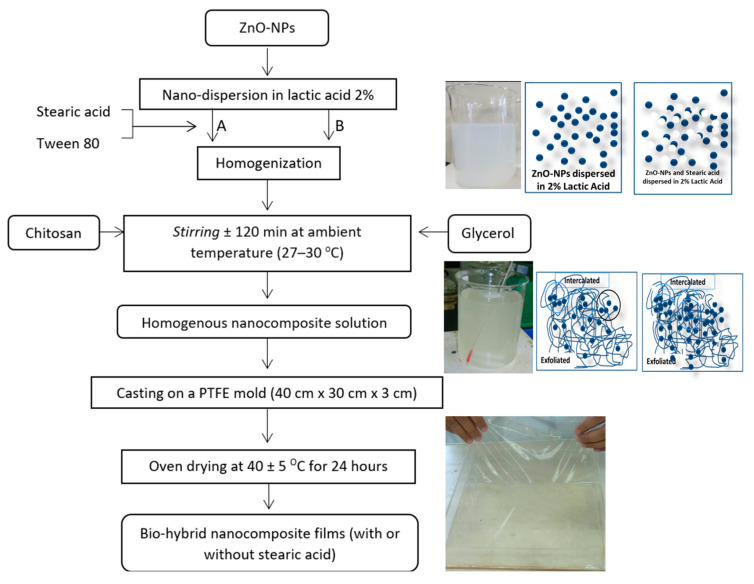
Flowchart of chitosan–ZnO-NPs nanocomposite film preparation: (A) with stearic acid, (B) without stearic acid.

Figure 1 illustrates the process flowchart used to produce chitosan–ZnO NPs biohybrid nanocomposite films with or without stearic acid. ZnO-NPs were first dispersed in lactic acid 2% with or without stearic acid with Tween 80 (Merck KGaA, Darmstadt, Germany) as emulsifier and homogenized using a high-speed homogenizer at 15,000 rpm for 2 min (POLYTRON Homogenizer from Kinematica AG, Malters, Switzerland) until a homogenous solution was obtained. Chitosan powder and glycerol chitosan polymer could act as capping agents for ZnO-NPs in this blending system to prevent stearic acid and ZnO-NPs from aggregating. The generated biohybrid nanocomposite solutions were then poured into a poly (tetrafluoroethylene) (PTFE) mold with dimensions of 40 × 30 × 5 cm^3^ to a solution depth of approximately 2 cm and dried in an oven drier at 40 °C for 24 h Before testing, the dry films were scraped from the casting surface and preconditioned in a desiccator at room temperature for two days at 75% relative humidity.

### 2.3. Optimization Using a Full Factorial Design and Statistical Analysis

Design Expert V13 (StatEase, Minneapolis, MN, USA) was used to build a full factorial design with multilevel categories for generating experimental runs, analyzing data (ANOVA and fit statistics with α = 0.05 for significant level), and optimizing and confirming the optimal formula. We defined two independent variables: ZnO-NP concentration at four levels (0%, 0.5%, 1%, 3% by weight of chitosan) and stearic acid (SA) amount at two levels (0% and 5% by weight of chitosan) with a constant amount of glycerol as a plasticizer and the addition of Tween 80 as an emulsifier when stearic acid was used. The amounts of ZnO-NPs and stearic acid used in this study refer to our previous studies with pectin and starch as biopolymer matrix [16]. While the responses (dependent variables) were water vapor permeability (WVP), percent elongation at break (% EB), tensile strength at break (TS), and antibacterial activity against Bacillus cereus, *Staphylococcus aureus*, and *Escherichia coli* indicated by inhibitory zone diameter. Further analyses include DSC thermal analysis, water activity, and SEM observation of film microstructure.

The mathematical model representing the response of a full factorial design is a linear polynomial model whose interactions are described by Equation (1).

Y_ijk_ = μ + A_i_ + B_j_ + (AB)_ij_ + ε_ijk_ (1)

where Y is the response and A, B, and AB are coded independent variables corresponding to the concentration of ZnO-NPs, the concentration of stearic acid, and their interaction, respectively. Indexes “i”, “j”, and “k” values are taken as i = 0%, 0.5%, 1%, and 3%; j = 0% and 5%; and k = 1, 2, 3 (number of replication).

### 2.4. Analysis of Biohybrid Nanocomposite Films

#### 2.4.1. Water Vapor Transmission Test Using Gravimetric Method

The water vapor transmission rate (WVTR) of the film was measured using a gravimetric technique (ASTM E-96) and the dry cup system. Desiccant (CaCl_2_) was placed in cups. Circular film samples (2.5 cm in diameter) were then placed on top of the cups and covered with wax to guarantee no water vapor leakage during the sample test. Before immersing the cup in a desiccator containing a saturated K_2_SO_4_ salt solution (97% RH), its initial weight was measured with an accuracy of 0.0001 g. The entire assembly was afterward weighed consistently every two hours for three days. We checked that no leakage occurred such that the whole increase in sample mass can be attributed to water vapor transmission. A graph depicting the relationship between weight gain and time is also drawn. WVTR is computed with Equation (2), given below:

(2) $$\mathrm{WVTR}=\;\frac{graph\;slope\;\left(\frac{g}{day}\right)}{sample\;area\;\left(m^{2}\right)}$$

WVTR units are g/(m^2^ day), measured at ∆RH = RH_out_ − RH_in_ = 97% RH at the temperature of 30 °C. All film samples for WVTR measurement were selected with the same thickness in the range of 33.2–33.7 μm.

#### 2.4.2. Mechanical Properties

A tensile testing machine was utilized to evaluate the films’ mechanical properties (Instron 4411, Norwood (Massachusetts), USA). In order to compute the cross-sectional area, the thicknesses of the film specimens were determined using a digital micrometer (Digimatic Micrometer, Mitutoyo, Japan) prior to testing. Each film specimen was placed between the grips of the tensile testing apparatus and tested with an initial grip spacing of 50 mm and a crosshead speed of 1 mm/s. The mechanical tests were replicated seven times. The tensile strength was calculated as the maximum force at break divided by the initial cross-sectional area of the film, and elongation at break (in %) was calculated as the delta length at break (∆L) divided by the initial gauge length.

#### 2.4.3. Antibacterial Activity with Agar Well Diffusion Assay

*Bacillus cereus* (ATCC 11778), *Escherichia coli* (ATCC 25922), and *Staphylococcus aureus* (ATCC 25923) were inoculated into 100 mL of Nutrient Agar (NA) with 0.2 mL containing 1 × 10^8^ CFU/mL, standardized with McFarland (standard 1). After solidification, five pieces were bored with a sterile cork borer to create a “well”. Five wells were filled with four treatments of biohybrid nanocomposite solution and one (in the center) with sterile water. The incubation was then completed for 24 h at 37 °C. Subtracting the exterior diameter from the inner diameter of the clear zone of the “well” yields the inhibition zone (cm).

#### 2.4.4. Scanning Electron Microscopy

Scanning electron micrographs were obtained using a Jeol JSM-5600LV (JEOL, Akishima, Japan). Film specimens were mounted on an aluminum stub covered with double-sided carbon tape and sputter-coated with gold to enhance surface conductivity. All specimens were observed in SEM at 5 kV.

#### 2.4.5. Thermal Analysis by DSC

Using a differential scanning calorimeter (SHIMADZU DSC-60, Kyoto, Japan), the thermal properties of the films were measured. After conditioning a sample specimen, 10 mg-weighted samples were cut and sealed in aluminum pans. The analysis was conducted between −30 °C and 300 °C at a scanning rate of 10 °C/min with an empty pan as a reference sample.

#### 2.4.6. FTIR Spectroscopy Analysis

FTIR spectra of chitosan and biohybrid nanocomposite films with and without SA were recorded using a Fourier-transform infrared spectrophotometer (Spectrum One FT-IR spectrometer, Perkin Elmer, Waltham, MA, USA) in the frequency range of 400–4000 cm^−1^. Sample films with thicknesses of around 20–30 μm were used for measurement. Each sample was scanned 16 times.

#### 2.4.7. Water Activity

Water activity was measured using the a_w_ meter (Shibaura WA–360, Saitama, Japan). Prior to measurement, the instrument was first calibrated using a saturated salt solution of NaCl. The recording was performed on the a_w_ value and temperature at the time of measurement.

## 3. Results and Discussion

### 3.1. Optimization Process with Full Factorial Design

#### 3.1.1. Number of Runs and Multiple Responses for the Optimization

Design-Expert^®^ software (DX13) offering a “Multilevel Categoric” option, which is known as a “general or conventional factorial” [22], is used in the present study. Design of the experiment by using full factorial facilitates the simultaneous assessment of the impact of using ZnO-NPs and SA as well as their interaction effects on the targeted responses. Table 1 presents the total 24 runs obtained from the full factorial design (4 × 2 with three replications). The order of run was randomized to optimize the process variable together with the experimental and the dependent variables, i.e., WVTR, %EB, TS, and antibacterial activity.

#### 3.1.2. Analysis of Variance (ANOVA) and Independent Variables’ Effect on Multiple Responses

The results of variance analysis (ANOVA) are presented in Table 2. It shows that the *p*-values of the factorial model for all response parameters are less than 0.01%, indicating that all models are valid. Table 2 also shows the main effect of Factors A (%ZnO-NPs) and B (%SA) and their interaction (AB). All responses with *p*-values less than 0.01% indicate significant effects of factors and their interaction. In the case of tensile strength, there was no significant interaction effect between ZnO-NP and SA (*p* = 0.4524). Additionally, Table 2 indicates that all the predicted R² of the responses are in reasonable agreement with the adjusted R²; i.e., the difference was less than 0.2, indicating that the fit statistics and all responses are qualified for the optimization process. Moreover, concerning the adequate precision, when measuring the signal-to-noise ratio (S/N ratio), a ratio greater than 4 is desirable. The current study led to ratios of 43.4378, 23.1187, 25.5774, 18.1951, 8.8458, 17.788 for response WVTR, %EB, TS, *B. cereus*, *S. aureus*, and *E. coli*, respectively, indicating an adequate signal. This confirms that each model of all responses can be used to navigate in the design space or qualified to be included in the optimization stage.

**Figure 2:**
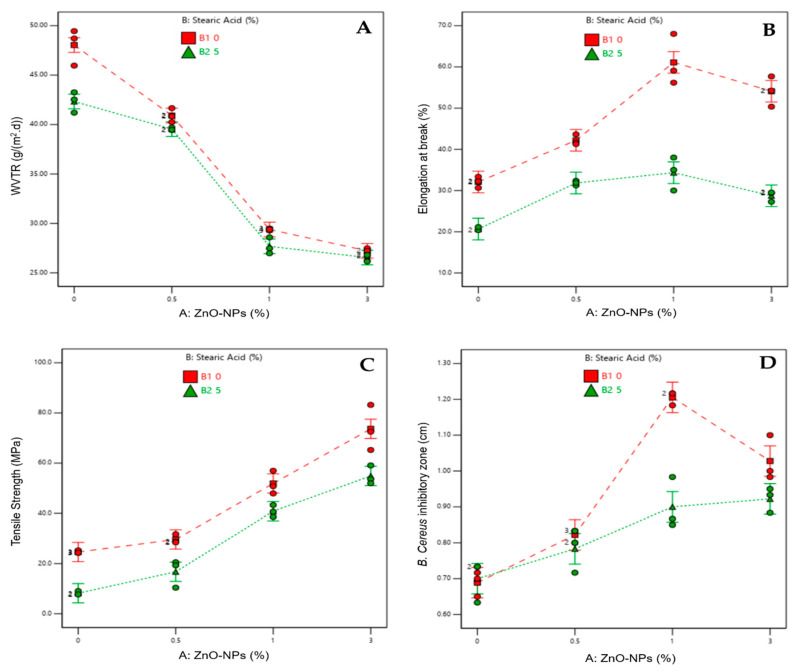

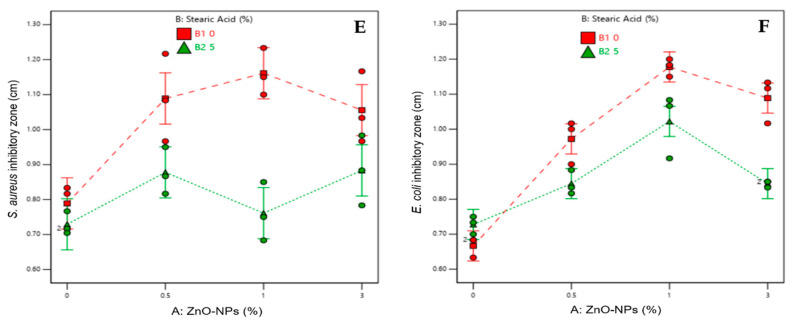
Interaction effect of ZnO-NPs and SA: (**A**) WVTR; (**B**) % elongation at break; (**C**) tensile strength; (**D**–**F**) antibacterial against *B. cereus*, *S. aureus*, and *E. coli*, respectively.

Figure 2 depicts the interaction effect of ZnO-NPs and SA on multiple responses: WVTR; % EB; Tensile Strength; and antibacterial activity against *B. cereus*; *S. aureus*; and *E. coli*. Except for tensile strength, Figure 2 indicates the significant effect of the interaction between ZnO-NPs and SA. In Figure 2C, the dashed red lines (representing 0% SA) and the solid green line (representing 5% SA) are nearly parallel, indicating no significant interaction. However, in Figure 2A,B,D–F, the dashed red lines and solid green lines are far from parallel, indicating a significant interaction between ZnO-NPs and SA.

Figure 2 reveals that the addition of SA has a favorable effect on the reduction of the WVTR value, which increases the water vapor barrier, but a negative effect on the other responses, i.e., a decrease in elongation at break, tensile strength, and antibacterial activities. Fortunately, the addition of ZnO-NPs has favorable effects on all responses, including a decrease of WVTR (Figure 2A), and an improvement of elongation at break (Figure 2B), tensile strength (Figure 2C), and antibacterial activities as well (Figure 2D–F).

##### Main Factor and the Interaction Effects on WVTR

With the addition of ZnO-NPs, lower WVTR values were obtained, as shown in Figure 2A. The lowest WVTR value is seen with the addition of 3% ZnO-NPs, which is 27.23 g/(m^2^.day). However, this WVTR value was not significantly different from the WVTR value with 1% ZnO-NPs (*p* > 0.05). Therefore, 1% ZnO-NPs is sufficient for generating films with excellent moisture barrier properties. Regarding the addition of SA, WVTR values were reduced when compared to similar formulations without stearic acid. Nevertheless, the reduction in WVTR values was not significantly different between formulations containing SA and those without SA (*p* > 0.05).

##### Main Factor and Interaction Effects on Mechanical Properties

The mechanical properties of films are illustrated in Figure 2B,C. It is noticed that when ZnO-NP content increased, the film’s tensile strength increased (Figure 2C). The addition of ZnO-NPs will fill the chitosan polymer matrix structure, enhancing the film’s integrity. Adding up to 3% ZnO-NPs to the biohybrid nanocomposite solution provided the strongest film in terms of tensile strength, elasticity, and strength for use as food packaging material. Unfortunately, the addition of SA to biohybrid nanocomposite films had unfavorable effects on film quality. The addition of SA reduces the film’s tensile strength compared to identical formulations without SA. The addition of SA may hinder the penetration of ZnO-NPs into the chitosan polymer matrix structure, resulting in a film with lower integrity than a film without SA. Based on an ANOVA analysis with a level of confidence of 5%, it was determined that the addition of SA led to a significant difference in the tensile strength of the film without the addition of SA. This demonstrates that the presence of SA has a negative effect; i.e., it may disrupt the films’ integrity.

Regarding the elasticity of films, as assessed by the %elongation (Figure 2B), the use of ZnO-NPs as a nanofiller for chitosan seems to have limitations. The %EB value increased with the addition of ZnO-NPs up to 1%, then dropped with the addition of 3% ZnO-NPs. This indicates that the formula with 1% ZnO-NPs may produce the highest %EB. The inclusion of SA tends to reduce the %EB values of nanocomposite films, as seen in Figure 2B. As for tensile strength, the presence of SA possibly created nonhomogeneous mixtures of chitosan–ZnO-NPs biohybrid nanocomposite films and weakened the resulting films’ integrity.

##### Main Factor and Interaction Effects on Antibacterial Activity

Figure 2D–F depict the antibacterial activity of biohybrid nanocomposite films produced using the “well method”. This investigation was conducted to test the antibacterial activity of chitosan-based biohybrid nanocomposite formulations as a function of ZnO-NP and SA concentrations. *Bacillus cereus* (Gram+), *Staphylococcus aureus* (Gram+), and *Escherichia coli* (Gram−) were the pathogenic bacteria tested. Due to differences in gram-positive and gram-negative bacteria cell walls, biohybrid nanocomposites are effective as antibacterial agents for both types of bacteria.

Gram-positive cell walls contain teichoic acid with a peptidoglycan (PG) thickness between 20 and 50 nm. Gram-negative bacteria have a cell wall component that is more complicated in terms of structure and chemistry than gram-positive bacteria [23,24]. Gram-negative cell walls contain a thin PG layer and a protective outer membrane. Metal oxide nanoparticles are known to be toxic to gram-positive bacteria [13], while chitosan solution’s antibacterial capabilities are more efficient against gram-negative bacteria [25]. The antibacterial activity of biohybrid nanocomposites is measured by the size of the clear zone, also known as the inhibitory zone. The wider the inhibitory zone formed, the greater the biohybrid nanocomposite antibacterial effectiveness. Theoretically, gram-positive bacteria (*B. cereus* and *S. aureus*) will be more easily affected by the antimicrobial properties of bionanocomposites because the cell wall of gram-positive bacteria does not have an outer membrane that can prevent the entry of hydrophobic compounds such as chitosan suspension into the cell. However, the sensitivity levels of bacteria do not depend only on the type of cell wall. Several other factors can affect the tolerance of bacteria to nanoparticles.

Antibacterial activity is one of the benefits of utilizing ZnO-NPs for film packaging. ZnO-NPs contribute to antibacterial properties to the active packaging via photon-induced production of reactive oxygen species (ROS) and the release of Zn^2+^ ions. ZnO-NPs can be applied to food surfaces in contact with pathogenic bacteria, resulting in bacterial death [26]. Figure 2D–F depict the results of the antibacterial activity of the chitosan-based biohybrid nanocomposite. With the addition of ZnO-NPs, the inhibition zone against gram-positive and gram-negative pathogenic bacteria increased. At a concentration of 3% ZnO-NPs, antibacterial activity was reduced. The expected result of incorporating SA into biohybrid nanocomposite films was not achieved. Figure 2D–F demonstrate that adding SA diminished the antibacterial activity of biohybrid nanocomposite films. Even though Casillas-Vargas et al. [21] found that SA has antibacterial action against gram-positive and gram-negative bacteria, it generates nonhomogenous mixtures that might alter the effect of ZnO-NPs.

#### 3.1.3. Optimization of the Multiple Responses

In the optimization process, the goal for independent variables was set to “in range”, and the importance level was set to 3 (default), while the goals of each response were set according to the research purposes (Table 3), i.e., WVTR was minimized to obtain maximum water vapor barrier; %EB was maximized to obtain maximum elasticity for avoiding crack during the application, and tensile strength was set to “in range” because application as edible film/coating does not require a rigid property. Consequently, the answer importance level was adjusted to 5 for all responses except tensile strength, which was set to 3.

Eight solutions were obtained using the Design Expert software, as shown in Table 4. The values of desirability range from 0 to 1, and the solution whose desirability values are close to 1 must be chosen as the optimal formula. Solution No. 1 is found to have the highest desirability value, which is 0.904. Thus, the formula achieved by combining ZnO-NPs with 1% by weight of chitosan without adding SA is considered optimal.

#### 3.1.4. Confirmation of the Optimum Formula

To confirm the optimal formula suggested by DX13, biohybrid nanocomposite films prepared with chitosan with 1% (*w*/*w*) ZnO-NPs without SA were produced six times, and the results are shown in Table 5. The average measurements were then compared to the DX13 prediction value shown in Table 6. All response data means fall between the 95% predicted interval (PI) low and 95% predicted interval (PI) high, indicating that the optimal formula was reached by utilizing 1% ZnO-NPs without SA. Despite the fact that the data mean for antibacterial activity against S. aureus was outside the expected interval, the actual value was greater than 95% PI high, so the research objective was achieved.

Based on the optimization and confirmation results, the formulation containing 1% (*w*/*w*) ZnO-NPs without the addition of SA should be the optimal formulation for use as an active film/coating. This observation was consistent with Figure 2 in that utilizing 3% (*w*/*w*) ZnO-NPs had a diminished beneficial effect. Furthermore, Jayasuriya et al. [13] showed that composite films containing 1% ZnO-NP (30 nm) would not induce cytotoxicity in cells, i.e., they would not be harmful to humans.

### 3.2. Films Morphology Observed by SEM

**Figure 3:**
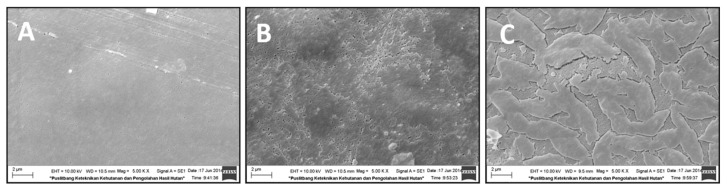
SEM Surface morphology: (**A**) control chitosan films; (**B**) biohybrid nanocomposite films made of chitosan+1%ZnO-NPs without SA; (**C**) biohybrid nanocomposite films made of chitosan+1%ZnO-NPs with 5%SA.

Figure 3 shows the surface morphology of chitosan films and chitosan – ZnO-NPs nanocomposite films. Figure 3A depicts the control film (chitosan alone), while Figure 3B,C exhibit biohybrid nanocomposite films formed from chitosan+ZnO-NPs with and without 5% SA, respectively. Both Figure 3A,B displayed a flat surface. In Figure 3B, a homogeneous structure with the appearance of ZnO-NPs as tiny white spots can be observed. ZnO-NP size is less than 100 nm, as measured using an SEM scale. This observation indicates that chitosan and ZnO-NPs were successfully fabricated into active biohybrid nanocomposite films. A similar result was reported by Shahvalizadeh et al. [27] that used gelatin as a biopolymer matrix. However, an uneven shape was observed when 5% stearic acid was added to the chitosan+1% ZnO-NPs matrix (Figure 3C). Figure 3C displays a rough surface, and it is probable that nanocomposite film structures contained micro-cracks. The addition of SA rendered the surface of chitosan+ZnO-NPs rougher and more heterogeneous, which explains the decreased effects on multiple responses, except on WVTR.

### 3.3. Water Activity

**Figure 4:**
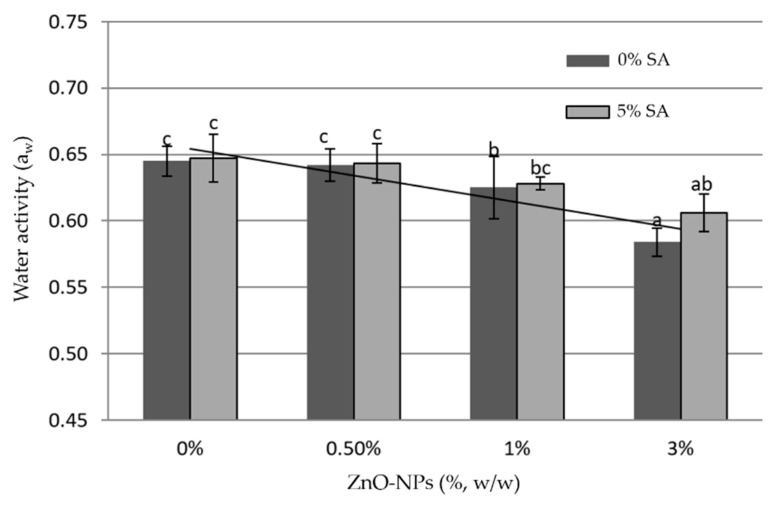
Water activity of biohybrid nanocomposite films (the error bars represent standard deviation and bars with different letters are significantly different based on Duncan’s multiple range test with *α* = 0.05).

Measurement of water activity (a_w_) aims to measure the ease with which microbes, such as bacteria, yeast, or mold, grow on this biohybrid nanocomposite film. The lower the a_w_ value, the more difficult it is for microbes to grow on it. Data measurements of a_w_ are presented in Figure 4, which shows the trend of reduction in a_w_ values with the addition of ZnO-NPs: the higher ZnO-NPs content is, the lower the a_w_ value is. The opposite effect was obtained by using SA: the presence of SA in the chitosan and chitosan+ZnO-NPs films increased the water activity value. For the purpose of food preservation, the higher a_w_ value is not expected because it is more favorable for microbial growth.

### 3.4. Thermal Properties

**Figure 5:**
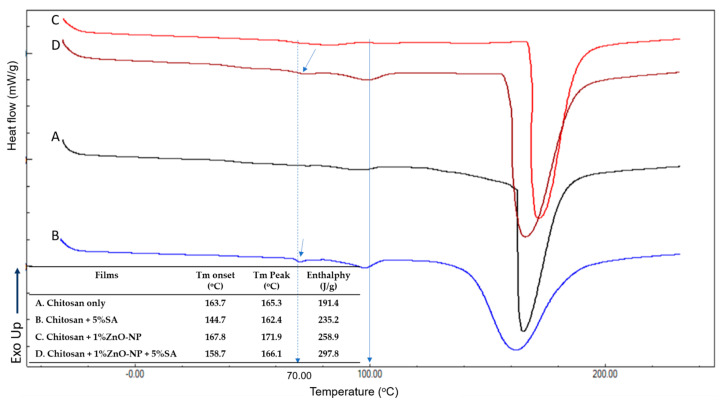
DSC thermogram of biohybrid-nanocomposite films: (A) chitosan only; (B) chitosan + 5%SA; (C) chitosan + 1%ZnO-NP; (D) chitosan + 1%ZnO-NPs+5%SA.

The thermal characteristics of the produced films were determined using DSC analysis. Due to its efficiency in revealing the thermal characteristics of chitosan, the approach of Erol et al. [28] was applied. The DSC thermograms of biohybrid nanocomposite films are depicted in Figure 5. All compositions exhibit an endothermic peak with a wide temperature range between 150 and 170 °C, which may correspond to the chitosan melting point. For the pure chitosan film and films containing 5% SA, there is a modest endothermic peak at about 100 °C that may be caused by the evaporation of water from the film. Qiao et al. [29] and Carvalho et al. [30] also reported the same observation. The addition of SA caused a larger endothermic peak at 100 °C, indicating more water evaporation from the film matrix. In the case of films formed from chitosan + 1%ZnO-NPs without SA, the endothermic peak at 100 °C was not detected, indicating any water evaporation. ZnO-NPs may bind water to generate Zn(OH)_2_, preventing the water from easily evaporating. This result revealed that the addition of ZnO-NPs decreased the water activity values, whereas the use of SA had the opposite effect and increased the a_w_. The films containing 5%SA exhibited an endothermic peak at around 70 °C, which is attributed to the SA melting temperature [31,32]. In Figure 5, it was noticed that the incorporation of ZnO-NPs enhanced both the onset and peak melting points of chitosan films, whereas the addition of SA decreased those values. Regarding enthalpy, the addition of ZnO-NPs and SA may increase the enthalpy values, indicating an increase in the film’s crystallinity.

Dong et al. [33] reported that the glass transition temperature (T_g_) of chitosan was determined by DSC to be between 140 and 150 °C. In the present study, the T_g_ of chitosan was not detected, which is in agreement with Akalin et al. [34]. This may be due to the rigid molecular structure of chitosan, which causes the T_g_ baseline step to be excessively broad. Cervera et al. [35] observed that many features, including crystallinity, amount of water, degree of deacetylation, and OH or amine groups in the chain of the macromolecule, could affect the appearance of chitosan’s glass transition.

### 3.5. FTIR Analysis

**Figure 6:**
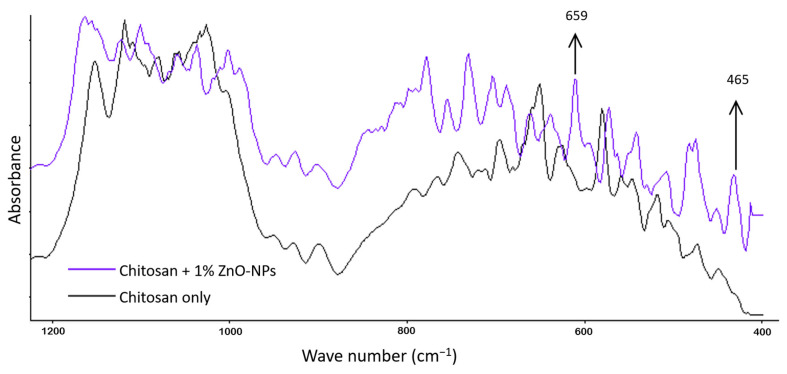
FTIR spectra of chitosan and chitosan + 1% ZnO-NP.

FTIR analysis was performed to determine whether the ZnO-NPs were successfully dispersed in the chitosan matrix. Using IR spectroscopy, Shuai et al. [36] analyzed hydrogen bonding and other interactions and the miscibility of polymer blends. Figure 6 illustrates the FTIR spectra of chitosan and chitosan with 1% ZnONP as processed in this study.

Figure 6 compares the FTIR of the chitosan-ZnO-NPs film to that of the chitosan film. In chitosan+ZnO-NPs film spectra, two additional peaks appear at 659 cm^−1^ and 465 cm^−1^, indicating the presence of amide groups and Zn-O, respectively. This result suggests that ZnO-NPs were successfully incorporated into the chitosan polymer matrix and that a specific interaction through hydrogen-bonding may occur between chitosan and ZnO-NPs. Abdelhady [37] achieved comparable FTIR results.

## 4. Conclusions

In this study, we achieved the fabrication of active biohybrid nanocomposite films based on chitosan, with improved multifunctional packaging properties. A full factorial design with multilevel categorical components and multiple functional food packaging characteristics responses has been applied to determine the optimal formula for preparing antibacterial chitosan-based biohybrid nanocomposite films. The incorporation of ZnO-NPs at 1% (by weight of chitosan) without using SA was confirmed to be the optimum formula. Although the inclusion of stearic fatty acids in the preparation of biohybrid nanocomposites films may raise the water vapor barrier, it caused a nonhomogeneous mixture with detrimental effects on tensile strength, elongation at break, and antibacterial activities. SEM analysis revealed that films made with SA were rough and likely cracked. Furthermore, observations on films without SA revealed that the size of ZnO-NPs in the chitosan matrix was in the nanoscale range (<100 nm). The DSC and FTIR analyses confirmed that interaction between chitosan and ZnO-NPs has occurred.

## Figures and Tables

**Table 1:** Full factorial design run matrix and the obtained multiple responses.

Run Number **	Independent Variables		Dependent Variables (Responses for Optimization)					
	ZnO-NPs	SA	WVTR	%EB	TS	Inhibitory Zone (cm)		
	(%, *w*/*w*)	(%, *w*/*w*)	(g/m^2^/day)		(kPa)	*B. cereus*	*S. aureus*	*E. coli*
1	1	5	28.59	38	40.6	0.98	0.75	1.08
2	3	0	26.98	54.2	72.5	1.10	1.03	1.13
3	0	5	43.26	20.4	7.8	0.73	0.77	0.75
4	3	5	26.8	29.5	52	0.93	0.88	0.85
5	1	0	29.48	68.0	50.8	1.22	1.15	1.15
6	0	0	48.7	32.3	24.3	0.72	0.82	0.68
7	3	0	27.23	50.3	83.2	1.00	0.97	1.12
8	1	0	29.29	59.1	56.9	1.22	1.23	1.18
9	0.5	0	40.24	41.3	28.5	0.80	0.97	1.00
10	1	5	27.48	35.0	43.3	0.87	0.85	1.07
11	1	0	29.39	56.2	47.9	1.18	1.10	1.20
12	3	0	27.48	57.7	65.2	0.98	1.17	1.02
13	0.5	5	39.44	31.3	20.5	0.83	0.82	0.82
14	0	0	45.96	33.3	24.3	0.65	0.72	0.63
15	0	5	41.2	20.4	9.1	0.63	0.72	0.73
16	0.5	5	39.7	32.4	10.4	0.72	0.87	0.88
17	0	0	49.45	30.6	25.3	0.70	0.83	0.68
18	0.5	0	40.83	41.7	31.7	0.83	1.22	0.9
19	1	5	26.98	30	38.6	0.85	0.68	0.92
20	0	5	42.53	21.2	7.7	0.73	0.70	0.70
21	0.5	0	41.66	43.6	28.7	0.83	1.08	1.02
22	3	5	26.15	29.4	59.1	0.88	0.98	0.83
23	0.5	5	39.47	31.9	19.3	0.80	0.95	0.83
24	3	5	26.69	27.3	53.6	0.95	0.78	0.85

Note: ** The order of run number was randomized using the software DX13.

**Table 2:** Results of ANOVA (full factorial model) and fit statistics.

Response Parameter	*p*-Value FFD Model	*p*-Value Factor A	*p*-Value Factor B	*p*-Value Inter. AB	Adj-R^2^ Model	Pred-R^2^ Model	Adeq Precision
WVTR	<0.0001 ****	<0.0001 ****	<0.0001 ****	0.0005 ****	0.9887	0.9824	43.4378
%EB	<0.0001 ****	<0.0001 ****	<0.0001 ****	0.0002 ****	0.9472	0.9173	23.1187
TS	<0.0001 ****	<0.0001 ****	<0.0001 ****	0.4524	0.9563	0.9316	25.5774
*B. Cereus*	<0.0001 ****	<0.0001 ****	<0.0001 ****	0.0002 ****	0.9174	0.8707	18.1951
*S. aureus*	<0.0001 ****	0.0008 ****	<0.0001 ****	0.0226 **	0.7565	0.6188	8.8458
*E. coli*	<0.0001 ****	<0.0001 ****	<0.0001 ****	0.0006 ****	0.9188	0.8729	17.788

Note: adj = adjusted, pred = predicted, adeq = adequacy; A = %ZnO-NP; B = % SA; Inter. = Interaction; *p*-value with **** = significant at *p* < 0.001; with ** = significant at *p* < 0.05; without ** = not significant.

**Table 3:** Constraints for the optimization process.

Variable Name	Goal	Lower Limit	Upper Limit	Importance
A: ZnO-NP	in range	0	3	3
B: Stearic Acid	in range	0	5	3
WVTR	minimize	26.15	49.45	5
%EB	maximize	20.41	68.02	5
Tensile Strength	in range	7.71	83.21	3
*B. cereus*	maximize	0.63	1.22	5
*S. aereus*	maximize	0.68	1.23	5
*E. coli*	maximize	0.63	1.20	5

**Table 4:** Solution of the optimized formula given by DX-13.

Solution No.	ZnO-NP	SA	WVTR	%EB	TS	*B. cereus*	*S. aereus*	*E. coli*	Desirability
**1**	**1**	**0**	**29.386**	**61.095**	**51.875**	**1.206**	**1.161**	**1.178**	**0.904**
2	3	0	27.228	54.093	73.651	1.028	1.056	1.089	0.757
3	0.5	0	40.909	42.191	29.637	0.822	1.089	0.972	0.474
4	1	5	27.681	34.333	40.838	0.9	0.761	1.022	0.414
5	3	5	26.55	28.731	54.873	0.922	0.883	0.844	0.41
6	0.5	5	39.538	31.829	16.749	0.783	0.878	0.844	0.322
7	0	0	48.037	32.079	24.614	0.689	0.789	0.667	0.11
8	0	5	42.33	20.657	8.199	0.7	0.729	0.728	0.076

**Table 5:** Response data of confirmation formula: ZnO-NPs 1% without adding SA.

Run	WVTR	%EB	TS	*B. cereus*	*S. aureus*	*E. coli*
1	28.52	63.4	51.9	1.21	1.32	1.30
2	31.17	62.6	52.3	1.38	1.32	1.09
3	30.75	61.4	52.4	1.28	1.31	1.10
4	29.84	66.2	49.9	1.17	1.30	0.99
5	31.79	62.7	48.0	1.26	1.28	1.12
6	30.28	65.2	50.6	1.34	1.39	1.23

**Table 6:** Confirmation result: actual vs. predicted value.

Response	Number of Replications	Prediction Value	95% PI Low	Data Mean of 6 Replications	95% PI High
WVTR	6	29.39	28.1	30.39	30.67
%EB	6	61.1	56.6	63.6	65.6
TS	6	51.9	45.2	50.8	58.5
*B. cereus*	6	1.21	1.13	1.27	1.28
*S. aereus*	6	1.16	1.03	**1.32**	1.29
*E. coli*	6	1.18	1.1	1.14	1.25

## Data Availability

Not applicable.
